# Clonality and genetic profiles of drug‐resistant *Mycobacterium tuberculosis* in the Eastern Cape Province, South Africa

**DOI:** 10.1002/mbo3.449

**Published:** 2019-02-23

**Authors:** Nolwazi L. Bhembe, Uchechukwu U. Nwodo, Anthony I. Okoh, Chikwelu L. Obi, Leonard V. Mabinya, Ezekiel Green

**Affiliations:** ^1^ SAMRC Microbial Water Quality Monitoring Centre University of Fort Hare Alice South Africa; ^2^ Molecular Pathogenesis and Molecular Epidemiology Research Group Department of Biochemistry and Microbiology University of Fort Hare Alice South Africa; ^3^ Academic and Research Division University of Fort Hare Alice South Africa; ^4^ Department of Biotechnology and Food Science Faculty of Science University of Johannesburg Doornfontein South Africa

**Keywords:** Beijing, *M. tuberculosis*, MIRU‐VNTR, Multidrug resistance, Spoligotyping

## Abstract

In this study, we investigated the diversity of drug‐resistant *Mycobacterium tuberculosis* isolates from families who own cattle in the Eastern Cape Province of South Africa using spoligotyping and mycobacterial interspersed repetitive‐unit‐variable number tandem repeat (MIRU‐VNTR) typing. The *Mycobacterium tuberculosis* was investigated using MIRU‐VNTR and the *Mycobacterium tuberculosis* families were evaluated using spoligotyping. Spoligotyping grouped 91% of the isolates into seven clusters, while 9% of the deoxyribonucleic acid (DNA) from TB isolates were unclustered from a total of 154 DNA used. Previously described shared types were observed in 89.6% of the isolates, with the Beijing family, SIT1, the principal genotype in the province, while the families T, SIT53 and X1, SIT1329 were the least detected genotypes. MIRU‐VNTR grouped 81% of the isolates in 23 clusters while 19% were unclustered. A combination of the VNTR and spoligotyping grouped 79% of the isolates into 23 clusters with 21% unclustered. The low level of diversity and the clonal spread of drug‐resistant *Mycobacterium tuberculosis* isolates advocate that the spread of TB in this study may be instigated by the clonal spread of Beijing genotype. The results from this study provide vital information about the lack of TB control and distribution of *Mycobacterium tuberculosis* complex strain types in the Eastern Cape Province of South Africa.

## 1 INTRODUCTION

*Mycobacterium tuberculosis* complex (MTBC) grounds eight million novel cases of tuberculosis (TB) and two million deaths every year. However, TB still remains a global epidemic with 8 million new‐fangled cases and 2 million deaths every year (Villemagne et al., 2012; WHO 2014). Although the World Health Organization has made strides in achieving the Millenium Development Goal of halting and reversing the incidence of TB, South Africa still has high incidences of TB, between 410, 000 and 520,000 (WHO 2014). The introduction of modern TB drugs in South Africa has decreased mortality and morbidity; however, the introduction of new and faster detection methods such as GeneXpert detected more cases because of its accuracy (Muller, 2001). The compounding factor for TB in South Africa is the advent and spread of drug‐resistant TB, especially multidrug‐resistant TB (MDR‐TB) [resistant to at least isoniazid (INH) and rifampicin (RIF)] and extensively drug‐resistant strains (XDR) TB [MDR‐TB with further resistance to any fluoroquinolone (FLQ) and to a minimum of one of the three injectable second‐line drugs, kanamycin (KAN), amikacin (AMK), and/or capreomycin (CAP)] (CDC 2006; WHO 2010). In 2006, the Eastern Cape Province of South Africa had a TB incidence of 705 of 100,000. In 2008, it had the solitary utmost MDR‐TB caseload in South Africa (WHO 2012). Deprived of adequate chemotherapy, TB could become increasingly mortal.

The World Health Organization reported that 274 patients were detected with XDR‐TB in October 2006 from the Eastern Cape Province, of which 23% died before treatment was initiated (WHO 2012; WHO/IUATLD, 2008). It further explains that, of the 206 patients who started treatment, 58.4% detected with XDR‐TB died. According to the National Health Laboratory Services, Eastern Cape had a total of 808 XDR‐TB between 2006 and 2010 (NDH 2011). Management of MDR and XDR‐TB strategy design hinge on the drivers of the epidemic, which are the prevalence and banquet of drug‐resistant strains as well as the understanding of the population structure (Said et al., 2012). TB drug‐resistant strains have been documented from previous studies in all the provinces in South Africa (Mlambo et al., 2008; Streicher et al., 2004; Warren et al., 1996). However, insufficient data have been reported from these studies. To the best of our knowledge, a comprehensive genotypic diversity of circulating MTBC strains is reported for the first time in the Eastern Cape Province of South Africa. Consequently, studies on the characterization of drug‐resistant strains are necessary for accurate assessments of population structure and to determine the common families of mingling drug‐resistant *M. tuberculosis* strains. Different genotypes occurring at different frequencies cause the global TB epidemiology (Bifani, Mathema, Kurepina, & Kreiswirth, 2000; van Soolingen et al., 1999).

The source and transmission patterns of drug‐resistant isolates can be traced using genotyping. Several methods such as IS*6110* restriction fragment length polymorphism (RFLP), spoligotyping, and mycobacterial interspersed repetitive‐unit‐variable numbers of tandem repeat (MIRU‐VNTR), have been used for molecular typing of *M. tuberculosis* (Cavusoglu, Turhan, Akinci, & Soyler, 2006; Cerezo et al., 2012; Mokrousov et al., 2004; Sola et al., 2003). IS*6110* RFLP typing is arduous, requires enormous quantities of DNA and has deprived discriminatory power on *M. tuberculosis* isolates with little or no IS*61 l0* copy number (Chaoui, Zozio, & Lahlou, 2014), although it is known as the orientation technique for genotyping of *M. tuberculosis* strains (Mathema, Kurepina, Bifani, & Kreiswirth, 2006; van Soolingen, 2001). These limitations of IS*6110* have been compensated by the development of PCR‐based methods such as spoligotyping and MIRU‐VNTR (Supply, Magdalena, Himpens, & Locht, 1997; Supply et al., 2006). Spoligotyping utilizes polymorphism in the direct repeat region, an organization belonging to a family of repeats called clustered repetitive interspersed palindromic repeats (CRISPRs) located in the genomes of bacteria and archaea (Kamerbeek et al., 1997; Pourcel, Salvignol, & Vergnaud, 2005). The method is practical and rapid in both clinical and molecular epidemiology and the results are uttered in an undemanding digital pattern, named and data based (Kamerbeek et al., 1997). However, when used unaided, spoligotyping tends to overrate the proportion of clustered strains. It is therefore required that spoligotype‐defined clusters be further defined using other methods such as MIRU‐VNTR (Sola et al., 2003). MIRU‐VNTR genotyping is a PCR‐based technique used to distinguish the number of tandem repeats at a specified genetic locus (Bifani et al., 1996). Most genotyping studies in South Africa were conducted in provinces with high multidrug‐resistant strains (MDRs) such as Western Cape (Warren et al., 1996), Gauteng (Hove, Molepo, Dube, & Nchabeleng, 2012), and KwaZulu‐Natal (Gandhi et al., 2014) and samples were collected from hospitals. However, little data are available from most of the provinces of South Africa, especially the cattle farmers’ in the Eastern Cape region. In this paper, we report on the clonality and genetic profiles of MTBC among drug‐resistant isolates from the Eastern Cape Province of South Africa as part of our bigger studies on reservoirs of antibiotic resistance determinants in South Africa.

## 2 EXPERIMENTAL PROCEDURES

### 2.1 Study population, Sample collection, and Ethical approval

A total of 6,000 suspected TB cases from households who own cattle in the Eastern Cape Province were considered in the study. This study is part of a larger research that investigated the prevalence of MTBC from cattle lymph nodes. The samples from this study were collected from people who own cattle and not from TB patients. The study was conducted at the Molecular Pathogenicity and Molecular Epidemiology Research Laboratory, University of Fort Hare, Alice. This project was approved by the University of Fort Hare Research Ethics Committee (UREC) and an ethical clearance certificate was issued; REC‐270710‐028‐RA Level 01. All individual identifiers have been detached or disguised, so the person(s) described are not identifiable and cannot be known through the details provided.

### 2.2 Sputum smear microscopy and Culture

Sputum smears were Ziehl–Neelsen (ZN)‐stained and acid fast bacilli (AFB) were examined under a bright field microscope. Similarly, all the collected sputum samples were also processed for in vitro drug susceptibility testing. Sputum samples were decontaminated using N‐acetyl L‐cysteine and neutralized with sodium hydroxide (Stroup et al., 2014). MTBC isolates were cultured on a Lowenstein–Jensen (LJ) slant followed by incubation at 37°C for at least 6–8 weeks.

### 2.3 Confirmation of *Mycobacterium tuberculosis* complex

DNA was extracted following the method by Yates, Drobniewski, & Wilson, 2002 and amplified using the Polymerase Chain Reaction (PCR) targeting the 240‐bp region of the *mpb64* gene using the outlined primer sequences F(460‐479) 5′‐TCCGCTGCCAGTCGTCTTCC‐3′ and R (700‐681) 5′‐GTCCTCGCGAGTCTAGGCCA‐3′ (Madhavan et al., 2000). The amplification was done in a 25‐μl reaction mixture, which consisted of 2.5 μl of 5× buffer (500 mmol/L potassium chloride, 100 mmol/L Tris chloride, 15 mmol/L magnesium chloride, gelatin 0.1%, pH 8.3), 100 ng each of primers, 200 mmol/L of each deoxyribonucleotide triphosphate, 1 U Taq DNA polymerase (Kapa Biosystems, South Africa), and 5 μl of DNA template. Nuclease‐free water was added to make the total volume to 25 μl. Amplification was executed using thermal cycler MyCycler^™^ (BioRad, Cape Town, South Africa). The protocol entailed of one cycle at 94°C for 5 min, 35 cycles of denaturation at 94°C for 1 min, annealing for 1 min at 55°C, and extension for 1 min at 72°C followed by one cycle of final extension at 72°C for 10 min (Madhavan et al., 2000).

### 2.4 Drug susceptibility testing (DST)

Confirmed MTBC isolates were tested for in vitro drug sensitivity testing for RIF, INH, and ethambutol (EMB) using the standard LJ proportion method as described earlier (Stroup et al., 2014; WHO 2009). Isolates were tested for resistance to RIF, INH, and ethambutol using concentrations of 40 μg/ml for RIF, 0.2 μg/ml for INH and 2.0 μg/ml for ethambutol. Briefly, the diluted suspension of isolates were inoculated onto LJ medium with and without drugs and incubated at 37°C. Results were read up to 42 days of incubation. An isolate was considered resistant to a given drug when growth of 1% or more as compared with the control was observed in drug‐containing medium. MTB H_37_Rv wild‐type strain (ATCC 27294) was used as a control for drug susceptibility testing (WHO 2009; Ali et al., 2015).

### 2.5 Genotyping and phylogenetic analyses

A spoligotyping commercial kit (Isogen Life Science B. V., Utrecht, The Netherlands) was used to perform spoligotyping (Kamerbeek et al., 1997) of the isolates. Spoligotypes in this study were assigned shared international types (SIT) numbers and genotypic cluster designations by comparing with the SITVIT4 database. MIRU‐VNTR typing was performed using 12 MIRU‐VNTR loci described elsewhere (Kamerbeek et al., 1997). NJ‐tree was constructed for combined spoligotypes and MIRU‐VNTR observed in our study using the MIRU‐VNTR*plus* web application (http://www.miru-vntrplus.org/MIRU/index.faces) (Weniger, Krawczyk, Supply, Niemann, & Harmsen, 2010).

### 2.6 Data analysis

**Figure 1:**
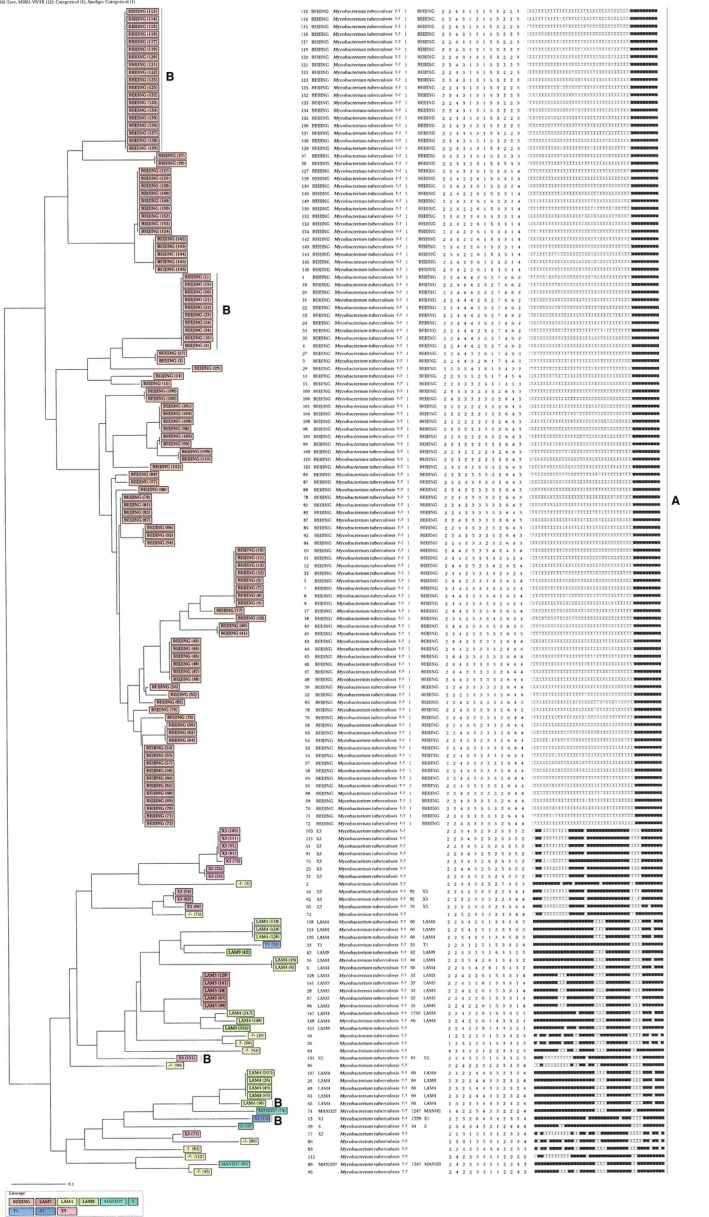
Relationship of spoligotypes of human DNA isolates from the Eastern Cape. The presented patterns were generated using the MIRU‐VNTR *plus* web application (http://www.miru-vntrplus.org/MIRU/index.faces. A  =  INR + RIF resistance; B  =  INH + RIF + EMB resistance

The spoligotyping results were entered in an Excel sheet as an octal format and binary code representing a positive or negative hybridization result. All spoligotype binary formats were submitted to the SITVIT2 (http://www.pasteur-guadeloupe.fr:8081/SITVITDemo) (Lillebaek, Andersen, Dirksen, Glynn, & Kremer, 2003). Major spoligotyping‐based phylogenetic subtypes were allocated according to signatures provided in SITVIT2, defining *M. tuberculosis* major lineages based on spoligotyping data (Demay et al., 2012). These include specific signatures for *M. tuberculosis* complex members, as well as rules defining major lineages/sublineages for *M. tuberculosis* sensu stricto as described elsewhere (Demay et al., 2012). MIRU‐VNTRplus Database (http://www.miru-vntrplus.org) was used to compare the 12 MIRU‐VNTR patterns using the Levenshtein algorithm (also called Edit‐Distance). In our case, the distance was calculated between each of our 12‐loci pattern with all the patterns available online (*n* = 141) in the MIRU‐VNTRplus database. Genotyping data was analyzed phylogenetically by constructing an NJ‐tree (Figure 1).

### 2.7 Statistical analysis

The Hunter–Gaston Index (HGI) was calculated as described previously (Hunter & Gaston, 1998), which was used in the determination of the diversity of each MIRU‐VNTR locus of the isolates, and described by the following equation, where N is the total number of isolates in the sample population for a given locus, S is the total number of distinct repeat unit values identified for the locus, and n_*j*_ is the number of isolates having the *j*th value:

$$\text{HGDI}=1-\frac{1}{N(N-1)}\sum_{j-1}^{s}n_{j}\left(n_{j}-1\right)$$

Dendrogram was generated with the program available online at www.miru-vntrplus.org (Allix‐Béguec, Harmsen, Weniger, Supply, & Niemann, 2008; Weniger et al., 2010) with the Neighbor‐joining tree generated. Fully identical patterns of *M. tuberculosis* isolates from different patients were assigned to the same cluster. The clustering rate was defined as (nc–c)/n, where nc is the total number of clustered cases, c is the number of clusters, and n is the total number of cases in the sample.

## 3 RESULTS

### 3.1 Sputum smear microscopy, culture, confirmation, and DST

Out of 6,000 suspected TB cases investigated in this study, 200 were positive when examined under the microscopy. As compared to microscopic examination, the numbers of positive specimens when using culture method were 156, which is lower than the initial screening. The entire specimen that were positive on culture, were also confirmed positive by PCR. Hundred and fifty‐four (98.7%) confirmed isolates were resistant to both INH and RIF, 2 (1.3%) resistant to INH alone, and 32 (20.4%) of the MDR detected were resistant to EMB.

### 3.2 Genotyping profiles of the *M. tuberculosis* DNA

We observed 11 SITs containing (*n* = 138, or 88.5% of the isolates) isolates summarized in Table 1. Among the 11 SITs recorded, seven SITs (containing 121 isolates, 87.6%) matched a pre‐existing SIT in the SITVIT2 database, whereas four (2.3%) isolates did not match any SIT in the SITVIT2 database (Table 1). The largest cluster generated consisted of 119 strains, (77% of all isolates), corresponded to SIT1, a prototype of the family Beijing genotypic lineage in the SpolDB4 and SITVITWEB databases commonly found in diverse parts of the world including Southern Africa. The SIT1 cluster was followed by Orphan/X3 (*n* = 7 or 4.5% isolates). A total of 29 Beijing strains were resistant to all three tested anti‐TB drugs, together with 0.6% of LAM2, X3, and X1 families (Figure 1). Two isolates were excluded due to DNA shortage and nogrowth when subcultured to isolate DNA.

**Table 1:**
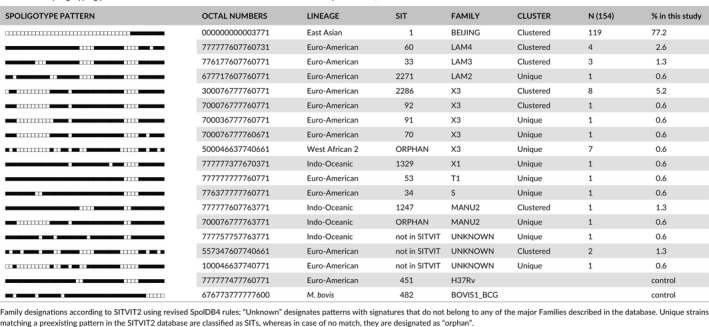
Spoligotyping patterns of 154 *M. tuberculosis* strains isolated in the Eastern Cape Province, South Africa

### 3.3 Discriminatory power of genotyping methods

The allelic variety among 12 MIRU‐VNTR loci (Table 2) differed from 0.022 to 0.787 in this study. Highly diverse (*h* > 0.6) (Hunter & Gaston, 1998), VNTR loci along with both Beijing and non‐Beijing isolates were found to be MIRU10, MIRU16, MIRU20, MIRU23, MIRU24, MIRU27, MIRU31, MIRU39. Nevertheless, centered on the value of allelic variety (*h* > 0.6) MIRU31 (*h* = 0.814), was more varied among non‐Beijing isolates. The biased power of each genotyping method is shown in Table 3. Spoligotyping showed a moderately biased power (HGI = 0.520) among 25 dissimilar types; spoligotyping recognized 10 distinctive isolates (6.5%) and 144 (93.5%) isolates grouped in seven clusters. The biggest cluster including 124 isolates was assigned to the Beijing family (ST1). Associated with spoligotyping, 12‐MIRUs alone recognized 25 distinctive isolates and 129 isolates in 23 clusters with a moderately biased power (HGI = 0.86). When used in combination with spoligotyping, 12‐MIRU achieved an HGI value of 0.951 (Figure 1).

**Table 2:** Number of occurrences of MIRU and allelic diversity for each locus

Allele locus	MIRU No	Allelic diversity (HGI)							
		No. of alleles	Beijing	Conclusion	No. of alleles	Non‐Beijing	Conclusion	No. of alleles	All
154	2	4	0.349	MD	3	0.462	MD	4	0.381
580	4	7	0.381	MD	4	0.729	HD	7	0.532
960	10	7	0.640	HD	5	0.613	HD	8	0.652
1644	16	6	0.682	HD	5	0.661	HD	8	0.678
2059	20	6	0.654	HD	6	0.822	HD	8	0.721
2531	23	7	0.642	HD	5	0.705	HD	7	0.658
2687	24	7	0.659	HD	6	0.802	HD	8	0.725
2996	26	6	0.605	HD	4	0.641	HD	6	0.617
3007	27	7	0.731	HD	5	0.527	MD	7	0.725
3192	31	8	0.756	HD	6	0.814	HD	8	0.787
4348	39	5	0.576	MD	4	0.691	HD	6	0.617
802	40	3	0.029	PD	2	0.041	PD	3	0.022

HGI, Hunter–Gaston index; PD, Poorly discriminatory (*h* < 0.3); HD, Highly discriminatory (*h* > 0.6); MD, Moderately discriminatory (0.3 ≤ *h* ≤ 0.6) (Sola et al., 2003; Zhao, Prakash, & He, 2012).

**Table 3:** Discriminatory power of spoligotyping and VNTR used alone and in association

Typing methods	Number of patterns	Number of clusters	Number of clustered isolates	Number of unique isolates	Size of clusters	Clustering Rate	HGI^a^
Spoligotyping	40	7	144	10	2–114	89	0.822
VNTR	51	23	129	25	2–124	69	0.940
Spoligotyping + VNTR	55	24	123	31	2–27	78	0.951

a HGI, Hunter–Gaston Index.

## 4 DISCUSSION

Previous studies (Marais et al., 2013; Said et al., 2012) described the clonal spread of genotypes of *M. tuberculosis* from different provinces of South Africa. However, an updated thorough analysis of the population structure of resistant *M. tuberculosis* isolates from the Eastern Cape Province, South Africa, was not available. As a result, this study is the first in the Eastern Cape Province to report about the population structure of resistant *M. tuberculosis*. This study, did not only attempt to map the population structure of drug‐resistant *M. tuberculosis* isolates from the Eastern Cape, but also differentiated the drug‐resistant isolates using 12‐locus MIRU‐VNTR. In this study, we collected sputum specimens from cattle farmers to trace the circulating MTBC strains that could be transmittable between humans and animals and socioeconomic activities practiced in this province. The Eastern Cape Province is one of the poorest provinces in South Africa, largely dependent on livestock farming (Katiyatiya, Muchenje, & Mushunje, 2014). As a result, there is a possibility of MTBC strains’ transmission between humans and animals; and hence, an investigation of genotypic strains in this province is highly imperative. We observed 99.4% MDR from MTBC isolates and a significant proportion (20.5%) of MDR isolates also resistant to EMB. Therefore, to minimize subsequent development of MDR strains, proper management of patients with TB is imperative. Early detection of TB is important in arresting further transmission of MDR‐TB clones, which is more expensive to manage owing to extended medication and high risk of death.

The predominant (69%) Beijing family represented by its isolated prototype SIT1 is abundant in our study. Both its elevated quantity of clonal spread and its majority among the novel patterns are appearances of the existing adaptive evolution of the South African genotype in this scenery (Streicher et al., 2004). In the Western Cape region, the Beijing genotype was vastly prevalent, and represented 36.5% of the drug‐resistant cases (Johnson et al., 2010). The Eastern Cape and Western Cape regions share a boarder on the western side of the Eastern Cape, and many residents of both Western Cape and Eastern Cape region have relatives in both regions. Strains might be carried from one region to another. Beijing family represented a high proportion of *M. tuberculosis* isolates causing ongoing TB transmission in Russia, Central Asia, and East Asia (Niemann et al., 2009; Wada, Iwamoto, & Maeda, 2009). Having 13% of the isolates globally and 50% of the isolates in Asia (Kim et al., 2001; Park, Bai, & Kim, 2000; Parwati, van Crevel, & van Soolingen, 2010), this family still has higher incidences (up to 92.59%) in Beijing and its immediate areas in China (Dong et al., 2010). Said et al. (2012) did not find any Beijing family in their study. The difference in the clonal spread of Beijing family in our study could be due to the difference in sampling size, time, and locations.

The LAM lineage (10.7%) was the subsequent most predominant in our study and was present in six out of 12 reported sublineages throughout the world (Demay et al., 2012). In contrast to other studies, the LAM family was reported at 22% of a total of 147 isolates in a study reported in Dar es Salaam, Tanzania (Eldholm, Matee, Mfinanga, Heun, & Dahle, 2006), while in Kenya, 11% of the families were LAM (Githui et al., 2004). Among the six sublineages present in South Africa, LAM4 was the most predominant (SIT60, 5.8% of strains). Different LAM subfamilies dominate in diverse coastal regions of Sub‐Saharan Africa (Pillay & Sturm, 2007; Viegas et al., 2010). LAM lineage is vastly prevalent in Latin American and the Caribbean's (Brudey et al., 2006); however, the clonal spread of LAM lineage (10.7%) found in the Eastern Cape is different; the spoligotype of LAM4 in our study (5.7%) with its prototype SIT60 and its variant 1750 (0.51%) were different from that of LAM9 (0.51%) with its prototype SIT42; altogether different from that obtained in Bogotá, Colombia (27.6%). We also found one strain in the LAM9 lineage that did not match any in the SITVIT2 and was considered as orphan. A study by Asiimwe, Ghebremichael, Kallenius, Koivula, and Joloba (2008) from Kampala showed proportions of LAM9 to be 2.6%. Out of the 12 sublineages that have been described globally for the LAM family (Brudey et al., 2006; Demay et al., 2012) a total of six sublineages were detected in our study. LAM3 in our study, represented by its prototype SIT33 was 2.6% which is higher than that obtained by Asiimwe et al. (2008) (1.7%). We also found LAM2 with its prototype SIT2271 (0.51%) in minority to all the LAM isolates. Of noteworthy, LAM6 was absent in our study. This was in contrast to the report elsewhere (Martins et al., 2013).

The X family of strains is defined by two associated features, a small number of IS*6110* copies and the lack of spacer 18 in the spoligotype (Sebban, Mokrousov, Rastogi, & Sola, 2002). The latter trait universal to at least three spoligotype shared types: ST119, ST137, and ST92 (Sebban et al., 2002). In our study, the X family with its variants was detected in 7.64%. This is not surprising as variants of this genotype family have been described in South Africa (Kim et al., 2001). Other noteworthy families identified in this study included the X family which was fairly distributed, T (mainly T1) and S families, which were among the minor families.

We also found the existence of a few ancestral Manu lineage strains (*n* = 4/193 or 2.07%) as clustered strains within our study sample. This lineage was first described as a new family in India in 2004 (Brudey et al., 2006) and later related strains in minute quantities were reported in a study from Madagascar (Ferdinand, Sola, Chanteau, & Sola, 2005). Rapidly later, it was cautiously subdivided into Manu‐1 (deletion of spacer 34), Manu‐2 (deletion of spacers 33‐34), and Manu‐3 (deletion of spacers 34‐36) sublineages, and suggested that it might denote an ancestral replica of main genetic group 1 strains (Brudey et al., 2006). Manu lineage strains were reported from Saudi Arabia (Al‐Hajoj et al., 2007), Tunisia (Namouchi et al., 2008), and in Egypt (Helal et al., 2009). In this study, 17 (8.8%) of the spoligo patterns could not be typed based on the current SpolDB4 database. This suggests the existing absence of information on the genetic diversity of *M. tuberculosis* strains from this region; therefore, appeals for additional clinical epidemiological studies in different provinces of South Africa to comprehend the genetic diversity of the TB epidemic in the country.

A high diversity among *M. tuberculosis* in this study was observed when MIRU‐VNTR genotyping technique was used. On its own, spoligotyping was the least discriminatory method when compared to MIRU‐VNTR typing and combined typing with spoligotyping and MIRU‐VNTR using Hunter–Gaston index (HGI). The peak allelic variety was witnessed for MIRU loci 10, 16, 20, 24, 27, 31. Loci 10, 16, 23, 26, and 40 were announced as the loci with the greatest allele polymorphisms, while loci 4, 20, 24, and 27 were the most poorly discriminated loci (Sola et al., 2003). However, in this study, MIRU loci 20, 24, and 27 were highly discriminative. This study was not on a population of TB patients, but it is based on cattle owners who had their animals investigated for TB. Hence, some degree of selection bias cannot be excluded. We could not conclude on aspects relating to drug resistance versus lineage in this study since the isolates used were not from a population study, but concentrated only on cattle owners, therefore introducing bias.

The evaluation of the stretch of *M. tuberculosis* families in this study might be difficult because of the shorter sampling period (2 months); however, it is still adequate in understanding the transmission patterns. Generally, clustering is indicative of continuing or current spread, while unique patterns indicate recrudescence events (Zozio et al., 2005). The spoligotyping and cluster analysis of 154 clinically isolated strains of MTBC showed that these 154 isolated strains had 60 genotypes. When spoligotyping is utilized together with MIRU‐VNTRs, the two methods are very discriminatory, supplying information for both the epidemiological and phylogenic characteristics of tubercle bacilli (Supply et al., 2006). In our study, the discriminating power of spoligotyping and MIRU‐VNTR separate were lower than the discriminating powers of both methods combined. Using the combined methods, the clustering rates were at 67%, whereas spoligotyping alone identified 87% clustering and MIRU alone identified 58.5% clustering. This suggests that each method alone can overestimate or underestimate clustering. Therefore, spoligotyping combined with MIRU‐VNTR can easily discriminate more isolates. The low level of diversity and the clonal spread of drug‐resistant *M. tuberculosis* isolates suggest that the spread of TB in this study may be instigated by the spread of Beijing genotype. A clearer picture of the spread and persistency of Beijing genotype in the Eastern Cape will be obtained once all isolates, susceptible and resistant, are investigated. However, this research still represents an important contribution in the lack of TB control and distribution of MTB strain types in the Eastern Cape Province. We conclude that spoligotyping and MIRU‐VNTR typing technique assists in widespread access and plays an imperative role in the epidemiological research of MTBC.

## CONFLICT OF INTEREST

No conflict of interest declared by authors.
